# Developmental localization and the role of hydroxyproline rich glycoproteins during somatic embryogenesis of banana (*Musa *spp. AAA)

**DOI:** 10.1186/1471-2229-11-38

**Published:** 2011-02-24

**Authors:** Chunxiang Xu, Tomáš Takáč, Christian Burbach, Diedrik Menzel, Jozef Šamaj

**Affiliations:** 1 College of Horticulture, South China Agricultural University, Guangzhou, 510642 Guangdong, PR China; 2 Centre of the Region Haná for Biotechnological and Agricultural Research, Department of Cell Biology, Faculty of Science, Palacký University, 783 71 Olomouc, Czech Republic; 3 Institute of Cellular and Molecular Botany, University of Bonn, Kirschallee 1, 53115 Bonn, Germany

## Abstract

**Background:**

Hydroxyproline rich glycoproteins (HRGPs) are implicated to have a role in many aspects of plant growth and development but there is limited knowledge about their localization and function during somatic embryogenesis of higher plants. In this study, the localization and function of hydroxyproline rich glycoproteins in embryogenic cells (ECs) and somatic embryos of banana were investigated by using immunobloting and immunocytochemistry with monoclonal JIM11 and JIM20 antibodies as well as by treatment with 3,4-dehydro-L-proline (3,4-DHP, an inhibitor of extensin biosynthesis), and by immunomodulation with the JIM11 antibody.

**Results:**

Immunofluorescence labelling of JIM11 and JIM20 hydroxyproline rich glycoprotein epitopes was relatively weak in non-embryogenic cells (NECs), mainly on the edge of small cell aggregates. On the other hand, hydroxyproline rich glycoprotein epitopes were found to be enriched in early embryogenic cells as well as in various developmental stages of somatic embryos. Embryogenic cells (ECs), proembryos and globular embryos showed strong labelling of hydroxyproline rich glycoprotein epitopes, especially in their cell walls and outer surface layer, so-called extracellular matrix (ECM). This hydroxyproline rich glycoprotein signal at embryo surfaces decreased and/or fully disappeared during later developmental stages (e.g. pear-shaped and cotyledonary stages) of embryos. In these later developmental embryogenic stages, however, new prominent hydroxyproline rich glycoprotein labelling appeared in tri-cellular junctions among parenchymatic cells inside these embryos. Overall immunofluorescence labelling of late stage embryos with JIM20 antibody was weaker than that of JIM11. Western blot analysis supported the above immunolocalization data. The treatment with 3,4-DHP inhibited the development of embryogenic cells and decreased the rate of embryo germination. Embryo-like structures, which developed after 3,4-DHP treatment showed aberrant non-compact epidermis with discontinuous ECM at the outer surface as well as much less immunolabelling with the JIM11 antibody. This treatment also decreased the plant regeneration capacity in embryogenic banana cultures. Finally, immunomodulation of surface hydroxyproline rich glycoproteins by co-culture of embryos with the JIM11 antibody resulted in a much lower germination capacity of these embryos.

**Conclusions:**

These results suggest that hydroxyproline rich glycoproteins play an important developmental role, especially in the process of regeneration and germination of embryos during plant regeneration *via *somatic embryogenesis. Proper content and localization of hydroxyproline rich glycoproteins seem to be essential for the formation and regeneration of banana somatic embryos.

## Background

Plant cell wall and the cytoskeleton control plant polarity and morphogenesis [1,2]. They determine cell shapes and control the fate of cells during cell differentiation. To better understand mechanisms, which regulate plant polarity and morphogenesis, it is very important to get a deeper knowledge about the functional architecture of the cell wall during cell shape acquisition and cell differentiation. Somatic embryogenesis requires strict spatio-temporal control over cell division and elongation/differentiation [3-5]. The polarity within the embryo is established through the precisely controlled cell division pattern of embryogenic cells (ECs) and elongation of supporting suspensor-like and callus cells. The cell wall appears to play an essential structural role during somatic embryogenesis [6,7].

Cellulose, hemicelluloses, pectin polysaccharides and structural proteins have been considered as the most abundant cell wall components. The major classes of cell wall proteins are arabinogalactan-proteins (AGPs), hydroxyproline-rich glycoproteins (HRGPs), proline-rich proteins (PRPs) and glycine-rich proteins. Extensins represent a well studied sub-family of HRGPs [8]. They have been implicated in nearly all aspects of plant growth and development including cell division and differentiation [9,10]. Some extensins were also proposed to be involved in the plant response to biotic [11-14] and abiotic stresses [11,15]. Additionally, extensins were implicated to have a role in the development of zygotic embryos in maize (*Zea mays *L.), *Arabidopsis *and tobacco (*Nicotiana tabacum*) [16-18]. To gain deeper insight in the possible functions of HRGPs in somatic embryogenesis, it is very important to localize them, and to study their biological function during somatic embryo development. However, to our knowledge, there are no reports about HRGP localization and function in somatic embryos of higher plants.

Antibodies represent one of the most useful probes for the study of plant cell walls, on the biochemical as well as on the structural levels in light and electron microscopy [19]. Tremendous progress has been made in the precise determination of cellular and subcellular distribution of cell wall components using diverse polyclonal and monoclonal antibodies. Among them, JIM11 and JIM20 recognize specific arabinosylation motifs of HRGPs such as extensins and Solanaceous lectins [20,21]. These antibodies were successfully used to study the distributions of extensins during plant developmental processes, such as pericycle and vascular tissue development [9,20], zygotic embryo development [18] but also during plant-microbe interactions [22]. In the present study, developmental immunolocalization of JIM11 and JIM20 epitopes was performed during somatic embryogenesis of banana (*Musa *spp. AAA group).

To study function of HRGPs, two main methods have been used to alter their content in the cell wall. The first one is a transgenic approach, which has been employed to study the effect of changes in HRGP gene expression level on the plant phenotype [23-26]. The other one is to use chemicals such as 3,4-DHP (3,4-dihydroxy-L-proline) which inhibits biosynthesis of HRGPs. Most of the proline (Pro) residues in HRGPs are hydroxylated by prolyl hydroxylases and the resulting hydroxyproline (Hyp) residues serve as major sites for O-glycosidic oligosaccharide decoration [27]. Thus, 3,4-DHP, as a potent inhibitor of prolyl hydroxylase, has been used to alter HRGPs in plant cell walls and thereafter to study the biological function of extensins [18,28-30].

In the present study, embryogenic cultures of banana were treated with 3,4-DHP or HRGP epitopes were immuno-modulated with the JIM11 antibody to affect HRGPs in the cell wall and to test the biological function of these glycoproteins during somatic embryo development.

## Results

### Expression pattern of HRGPs in ECs, NECs and somatic embryos of different developmental stages

**Figure 1:**
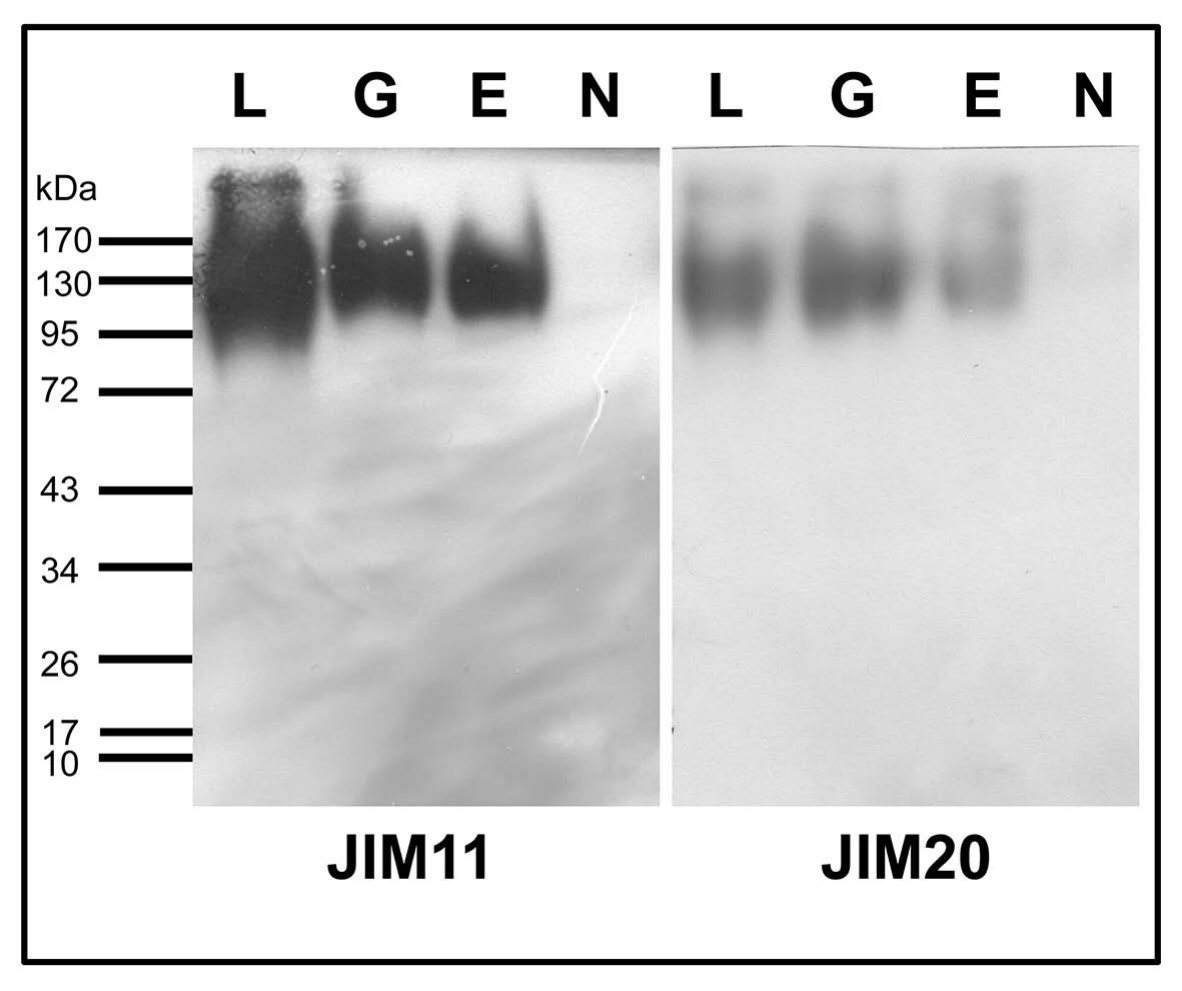
**Western blot analyses of JIM11 (a) and JIM20 (b) epitopes expression during somatic embryogenesis of banana**. E:embryogenic cells; N:nonembryogenic cells; G:embryos at globular stage; L:embryos at late stages.

Immunoblots were used to detect the expression of HRGPs in NECs, ECs and embryos of different developmental stages by using monoclonal anti-HRGP antibodies JIM11 and JIM20 (Smallwood et al. 1994). As shown in Figure 1, there was negligible signal of JIM11 in NECs, while there was a strong signal in ECs, globular embryos and especially in late-stage embryos (Figure 1a). The JIM20 epitope was moderately expressed in embryogenic tissues while only very low expression was detected in NECs (Figure 1b). The signal of JIM20 in later-stage embryos was relatively weaker than that found in embryos at globular stage (Figure 1b). There were two major HRGP bands in ECs and tissues with molecular weight of around 220 and 125 kDa, respectively (Figure 1). These immunoblot results were corroborated by immunofluorescence labelling data.

### Immunolocalization of HRGPs in NECs, ECs and somatic embryos of different developmental stages

**Figure 2:**
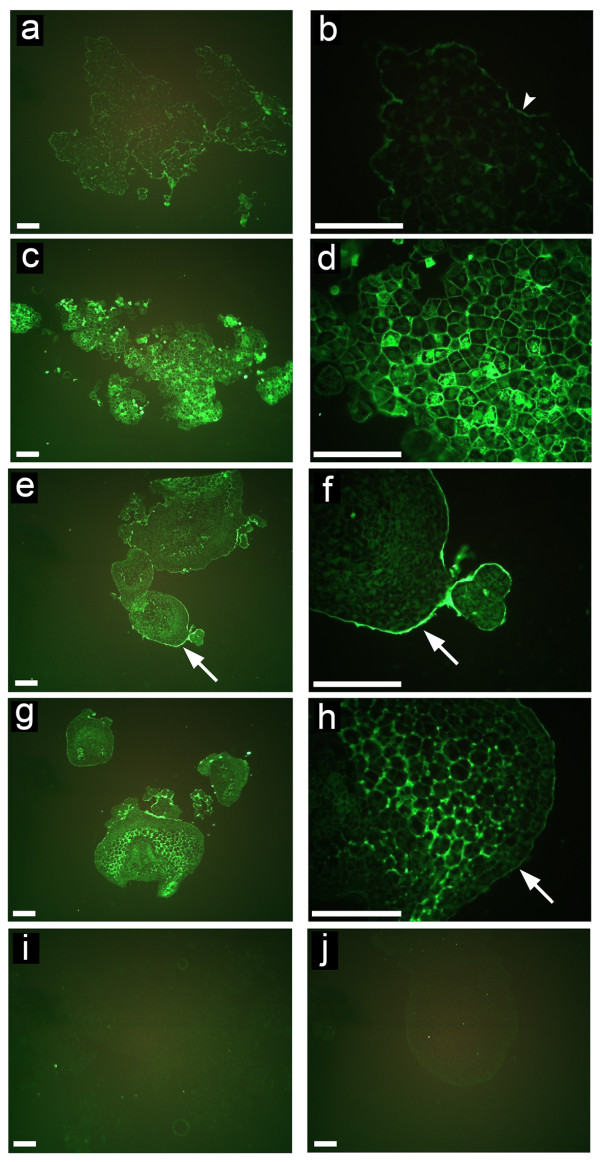
**Developmental immunofluorescence localization of JIM11 epitope during somatic embryogenesis of banana**. **(a) **Nonembryogenic cells showing signal located mainly at the surface of cell aggregates. **(b) **Detailed view from Figure (a) with arrowhead pointing on the moderate JIM11 signal at the surface of cell aggregates. **(c) **Embryogenic cells with strong signal, especially in the cell wall and cytoplasm around the nucleus but without signal on the surface of cell groups in many cases. **(d) **Detailed view from Figure (c) showing strong JIM11 signal in ECs. **(e) **Proembryos and globular embryos showing epidermis with strong surface fluorescence (arrow). **(f) **Detailed view from Figure (e) showing strong fluorescence in ECM covering epidermal cells (arrow). **(g) **Embryos at later stages. **(h) **Detailed view from Figure (g) showing strong signal in the tri-cellular junctions of cortical cells and moderate signal in the ECM at the surface (arrow). **(i) and (j) **Negative controls (labelled solely with secondary antibody) for ECs (i) and globular embryo (j). Bars, 100 μm.

**Figure 3:**
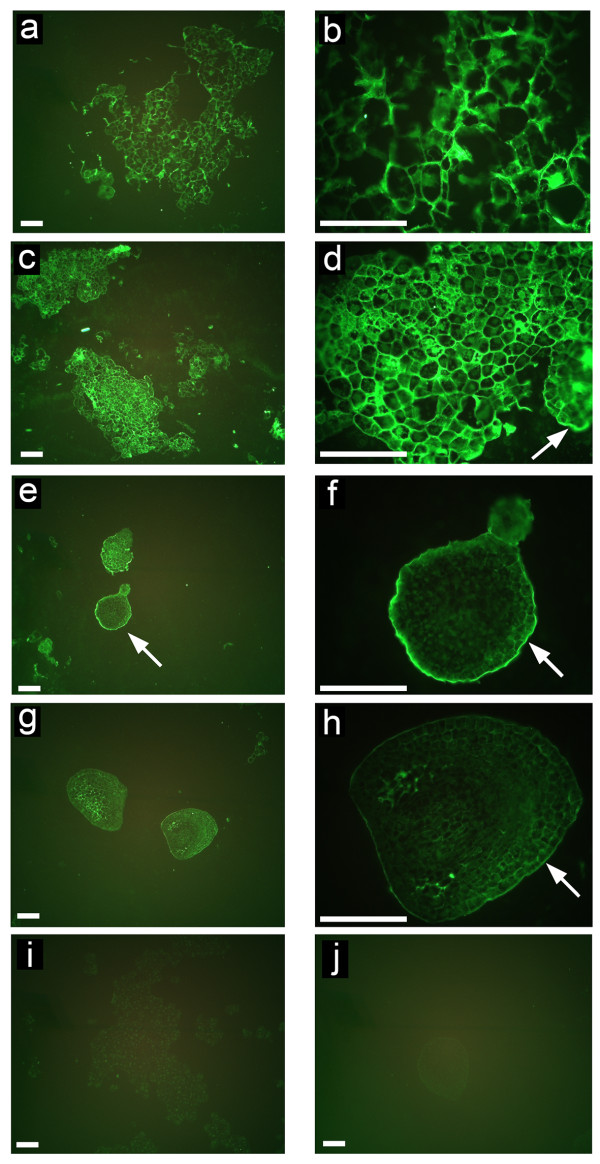
**Developmental immunofluorescence localization of JIM20 epitope during somatic embryogenesis of banana**. **(a) **Nonembryogenic cells. **(b) **Detailed view from Figure (a), showing strong signal mainly in the cell walls. **(c) **Embryogenic cells. **(d) **Detailed view from Figure (c) showing stronger fluorescence, especially in the cell walls and cytoplasm around the nucleus as well as at the surface of cell aggregates (arrow). **(e) **Proembryos and globular embryos showing very strong surface fluorescence (arrow). **(f) **Detailed view from Figure (e) showing strong fluorescence in ECM covering epidermal cells (arrow). **(g) **Embryos at later stages. **(h) **Detailed view from Figure (g) showing strong signal in the tri-cellular junctions of cortical cells and moderate signal in the ECM at the surface (arrow). **(i) and (j) **Negative controls (labelled solely with secondary antibody) for non-embryogenic cells (i) and pre-globular embryo (j). Bars, 100 μm.

NECs, ECs and somatic embryos at different developmental stages were labelled with monoclonal anti-HRGP antibodies JIM11 and JIM20. These results revealed that the fluorescence signal of JIM11 epitope was generally very weak in the NECs. Moderate fluorescence signal was found only at the surface of cell aggregates (Figure 2a and 2b). On the contrary, much stronger fluorescence was found in ECs, especially in the cell wall and cytoplasm around the nucleus, but mostly no signal was detected at the surface of cell groups (Figure 2c and 2d). In young pro-embryos, there was a very strong fluorescence layer at the cell surface but only moderate fluorescence inside the cells (Figure 2e and 2f). With the development of the somatic embryos, the fluorescence layer covering embryonic epidermis became thinner, however, new and strong fluorescence signal appeared in the cells within the embryo. Detailed study revealed that the JIM11 epitope was abundant in the cell walls and especially in the tricellular junctions of the inner cortical cells (Figure 2g and 2h). The negative controls showed almost no labelling of ECs (Figure 2i) and somatic embryo (Figure 2j). When compared to JIM11, there was slightly stronger signal of JIM20 in NECs, which was mostly located in the cell walls (Figure 3a and 3b). The immunolabelling results of JIM20 in ECs and pro-embryos were similar to those of JIM11 (Figure 3c-f). Nevertheless, in comparison to JIM11 there was always relatively strong signal of JIM20 at the surface of EC groups (Figure 3d). Moreover, the JIM20 signal in late stage embryos was weaker than that of JIM11 (Figure 3g and 3 h). Again, negative controls showed only very negligible unspecific signal (Figure 3i and 3j).

An overview of immunolabelling of JIM11 and JIM20 epitopes in different cell types and embryogenic stages is summarized in Table 1.

**Table 1:** The intensity evaluation of immunofluorescence labelling with JIM11 and JIM20 antibodies

Developmental stage	Cell types	Antibody	
		**JIM11**	**JIM20**

NECs	Inner cells	±	++
	Outer cells	+	++

ECs	Inner cells	++++	++++
	Outer cells	++++	++++

Proembryos and globular embryos	Epidermal cells	+++	+++
	Inner cells	+	+

Late embryos	Epidermal cells	+	+
	Subepidermal/cortex cells	+	+
	Cells around procambium	+++	++
	Procambium cells	±	±

NECs: non-embryogenic cells; ECs: embryogenic cells. Increasing intensity was evaluated as: ± (very weak), + (weak), ++ (middle), +++ (strong), ++++ (very strong).

### Effect of 3,4-DHP treatment and immunomodulation by JIM11 antibody on the growth, development and regeneration of somatic embryos

**Figure 4:**
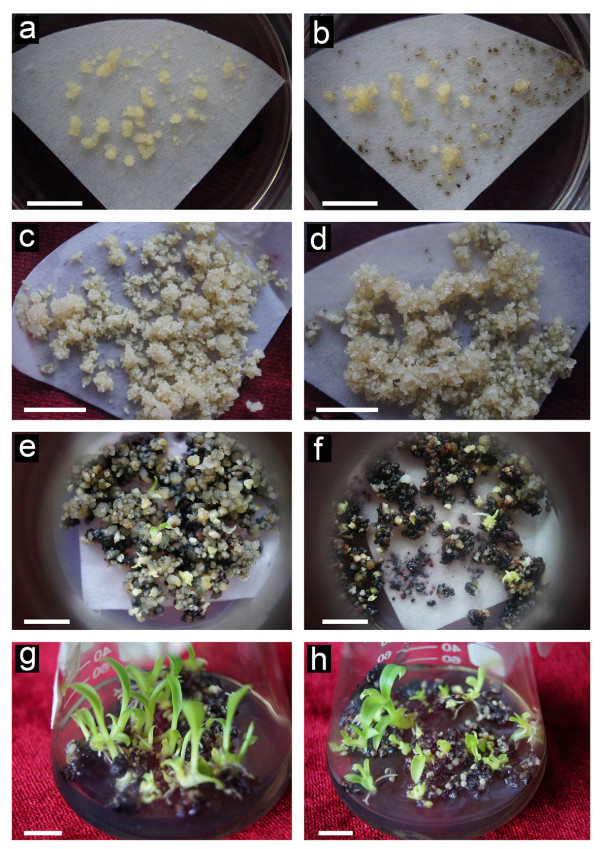
**The effect of 3,4-DHP on the development and germination of banana somatic embryos and on the plant regeneration**. Images **(a), (c), (e) **and **(g) **represent controls. Images **(b), (d), (f) **and **(h) **represent treatment with 200 μM of 3,4-DHP in RD1 embryo regeneration medium. **(a) **and **(b) **Embryo cultures one week after inoculation on RD1 medium showing many small black/necrotic cell aggregates resulting from 3,4-DHP treatment in the Figure (b). **(c) **and **(d) **Embryos 4 weeks after inoculation on RD1 embryo regeneration medium showing light brown embryos obtained on the embryo regeneration medium supplemented with 3,4-DHP in the Figure (d). **(e) **and **(f) **Embryo development 4 weeks after inoculation on RD2 medium for embryo maturation showing increased brown and black colour of 3,4-DHP treated plant material in the Figure (f). **(g) **and **(h) **Plant regeneration 4 weeks after inoculation on REG medium showing less regenerated plants after 3,4-DHP treatment in the Figure (h). Bars, 1 mm.

To target HRGPs/extensins more specifically, 3,4-DHP was added directly into RD1 embryo regeneration medium. One week after transfer of ECs on the RD1 medium supplemented with 3,4-DHP, many small cell aggregates showed necrosis (brown and black colour in Figure 4b), unlike to fully viable cell colonies in the control (Figure 4a). About two weeks later, some cells gradually recovered. At the end of culture on the RD1 medium, embryo-cultures were light brown (Figure 4d) while fresh weight was significantly reduced if compared to the control (Figure 4c, Table 2). Brown and black colour (indicating cell necrosis) in 3,4-DHP treated cultures increased on RD2 medium (Figure 4f). Simultaneously with this phenomenon, both embryo germination and plant regeneration capacity were significantly lower in 3, 4-DHP treated cultures as compared to the control (Figure 4g and 4h, Table 2).

**Table 2:** Effect of 3,4-DHP on somatic embryo development and plant regeneration

Treatment	Change in weight on RD1 medium	Number of embryos (×10^3^)/g regenerated on RD1 medium	Number of embryos (×10^4^)/g from ECs on RD2 medium	Number of plants (×10^3^)/g ECs on RD2 medium	Germination percentage of embryos (%) on RD2 medium
Control	20.21 ± 0.59 **	1.93 ± 0.18 *	3.89 ± 0.11	5.59 ± 0.13 **	14.05 ± 0.59 **
200 μM DHP	15.46 ± 1.87 **	2.45 ± 0.26 *	3.79 ± 0.46	3.18 ± 0.04 **	8.51 ± 0.23 **

ECs: embryogenic cells. The data in the table represent an average of four biological replicates ± standard deviation. A comparison of groups was conducted using a paired t-test of variance. Values marked with star were considered significant at P < 0.05 while values marked with two stars were considered significant at P < 0.01

**Figure 5:**
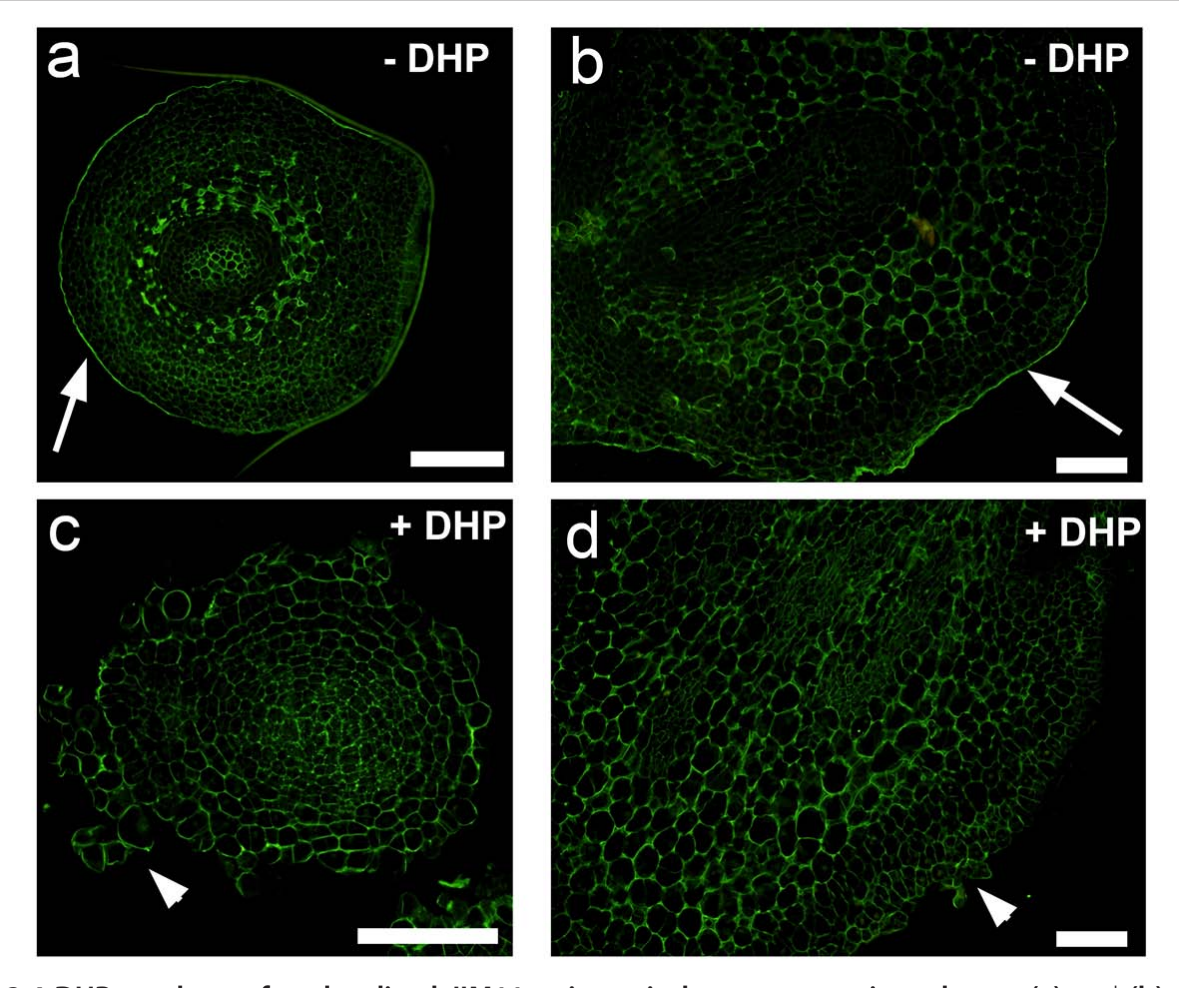
**The effect of 3,4-DHP on the surface localized JIM11 epitope in banana somatic embryos**. **(a) **and **(b) **Control embryos (arrows point to the regularly organized epidermis covered by ECM with strong JIM11 fluorescence. **(c) **and **(d) **Five-week-old embryos maintained on RD1 medium supplemented with 3,4-DHP. Arrowheads indicate disintegration of epidermis and formation of callus-like tissue at embryo surfaces. Note disruption of JIM11-positive ECM. Bars, 100 μm.

To evaluate an effect of 3,4-DHP on the distribution and localization of HRGPs in somatic embryos, immunolabelling with JIM11 antibody was carried out on embryos grown on RD1 medium supplemented with 3,4-DHP. Some of these embryos showed slightly less labelling with the JIM11 antibody when compared to the control. Most importantly, epidermis of embryos treated with 3,4-DHP was disorganized and the fluorescent layer representing ECM at the surface of these embryos disappeared when compared to the control (Figure 5).

**Figure 6:**
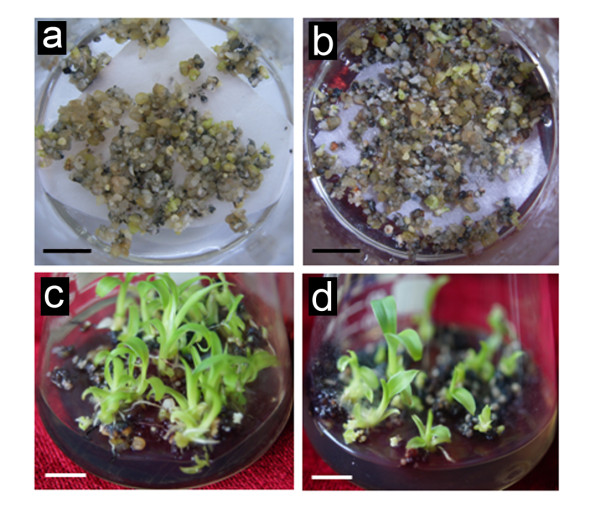
**The effect of immunomodulation with the JIM11 antibody on banana embryo germination and plant regeneration**. **(a) **and **(b) **Embryo cultures 4 week after inoculation on RD2 medium of the control (a) and after the treatment with JIM11 antibody (b). Note light brown colour of treated embryos in Figure (b). **(c) **and **(d) **Plant regeneration 4 weeks after inoculation on REG medium showing less regenerated plants after immunomodulation with JIM11 antibody in the Figure (d). Bars, 1 mm.

Finally, somatic embryos were surface-treated with JIM11 antibody to immunomodulate HRGP epitopes in the ECM. These embryos were subsequently transferred to RD2 and REG media for maturation and germination. At the end of culture on the RD2 medium, antibody-treated embryo-cultures were light brown to black while this was not the case with the control showing mostly white or yellow colour of embryos (Figure 6a and 6b). Subsequently, fewer plants were obtained from the same amount of antibody-treated embryos when compared to the control (Figure 6c and 6d, Table 3). Statistically significant differences were found between the control and antibody treatment, showing germination efficiency of 28.68 ± 3.52% and 19.60 ± 0.93%, respectively (Table 3).

**Table 3:** Effect of immunomodulation with JIM11 antibody on somatic embryo germination and plant regeneration

Treatment	Number of treated embryos	Number of regenerated plants	Germination percentage of embryos (%)
Control	364.38 ± 3.44	104.67 ± 13.54 *	28.68 ± 3.52 **
JIM 11 antibody	364.38 ± 6.86	71.33 ± 2.73 *	19.60 ± 0.93 **

ECs: embryogenic cells. The data in the table represent an average of four biological replicates ± standard deviation. A comparison of groups was conducted using a paired t-test of variance. Values marked with star were considered significant at P < 0.05 while values marked with two stars were considered significant at P < 0.01

## Discussion

HRGPs represent a major protein component of plant cell walls [8]. They are rich in hydroxyproline but also in serine, lysine, tyrosine, and valine residues, and they contain arabinose and galactose in the attached oligosaccharide chains [31-33]. Extensins represent a subfamily of HRGPs. In contrast to dicotyledonous plant species, the extensin subfamily of monocotyledonous plants is relatively simpler. They are rich in threonine or histidine rather than serine, and hence they are called threonine- or histidine-hydroxyproline-rich glycoproteins (THRGPs or HHRGPs) [32]. Moreover, extensins of dicots are highly glycosylated, contain 50-60% (w/w) of carbohydrate and form a left-handed polyproline II helix while extensins of monocots are less glycosylated and exist in a random coil conformation [32].

Monoclonal antibodies JIM11 and JIM20 recognize specific arabinosylation patterns of HRPGs such as extensins and *Solanaceous *lectins but not those of arabinogalactan proteins [20,21]. Since banana also contain lectins [34,35] it is possible that JIM11 and JIM20 antibodies recognize except extensins also these lectins. The JIM11 and JIM20 antibodies were used previously to study extensin, extensin-like and HRGP epitopes in diverse dicotyledonous plants [18,20,21] but also in green alga [36] and green seaweed [37]. In monocotyledonous species such as onion, JIM11 and JIM20 extensin epitopes were localized to rhizodermis, exodermis, endodermis, pericycle and phloem of primary root as well as to the root surface (Casero et al. 1998). Here, to our knowledge for the first time, the localization and function of JIM11 and JIM20 HRGP epitopes were studied during somatic embryogenesis of banana, a very important monocot fruit and crop.

In monocotyledonous maize, the mRNA of HRGP accumulates in young organs rich in dividing cells but it decreased in mature tissues [38]. Moreover, it showed a specific pattern of expression in immature embryos [39]. Further study revealed that the accumulation of this mRNA occurred early during cell differentiation and before acquisition of the final cell wall structure [40]. In the present study we showed that ECs of banana contained HRGP epitopes recognized by JIM11 and JIM20 antibodies. Thus, these epitopes might serve as good markers of embryogenic competence in ECs. During embryo development from ECs, the same JIM11 and JIM20 epitopes were abundant at the surface of proembryos and globular embryos. They were likely associated with the proper adhesion and monolayer formation of embryo epidermis. In late-stage embryos, however, the JIM11 and JIM20-positive signal was stronger in inner cortical and vascular tissues. We also showed that developmental distribution and subcellular localization of these surface-located HRGP epitopes were affected by 3,4-DHP treatment, which led to the disintegration of the ECM and disaggregation of the epidermis (resembling callus formation). Particularly important was finding that both immunomodulation with JIM11 antibody as well as treatment with 3,4-DHP negatively affected and reduced embryo formation and germination as well as plant regeneration capacity from banana somatic embryos. Altogether, these data suggest that developmentally regulated HRGP proteins are essential for development, germination and regeneration of banana somatic embryos. Similar results were recently reported by Zhang et al. [18] on tobacco zygotic embryo development. These authors suggested that extensins reacting to the same antibodies JIM11 and JIM20 play important roles in the cotyledon primordium formation, in the activity of the shoot apical meristem and in vascular differentiation during embryo development.

Although there are many differences between HRGPs and extensins of monocotyledonous and dicotyledonous plant species, there are still some similarities between them. There are few reports about similar localization of extensin epitopes in monocotyledonous and dicotyledonous plant species. For example, in rice (*Oryza sativa *L.), JIM12 and JIM20 antibodies raised against extensins from dicotyledonous plant species labelled the root tissues in the same pattern as the LM1 antibody [41] which was derived against extensins from rice [42]. Monocot barley and rice protoplasts contain JIM19 and JIM20 extensin-like epitopes [41,43], while there were both similarities and differences to the labelling pattern detected in dicot pea [37]. Here we show that JIM11 and JIM20 antibodies prepared against extracts from dicotyledonous plants such as carrot and pea, respectively [20,21] could recognize HRGPs in banana.

Interestingly, synthetic decapeptide matching the C-terminal sequence of inversion-specific glycoprotein (ISG), a HRGP from algae closely related to the extensins from higher plants, was able to disaggregate alga into individual cells [44] and this ISG was likely involved in the early processes of ECM biogenesis. Little is known about chemical composition, biogenesis and function of ECM at the surface of somatic embryos [45,46], especially in monocot plant species. In maize, the ECM contains AGP and pectin epitopes [6,7]. Here, we found, to our knowledge for the first time, JIM11 and JIM20 HRGP epitopes in the ECM covering outer surface of banana somatic embryos while this ECM was disrupted by treatment with 3,4-DHP.

## Conclusions

Immunoblot and immunofluorescence analyses revealed two HRGP epitopes JIM11 and JIM20 in ECs and in various developmental stages of banana somatic embryos. Interestingly, these epitopes were found also in the ECM at the surface of embryogenic cells. Treatment with extensin inhibitor 3,4-DHP depleted surface-localized JIM11 and JIM20 epitopes and also disrupted ECM. Additionally, both treatment with 3,4-DHP and immunomodulation with JIM11 antibody showed similar negative effects on the embryo development, germination and plant regeneration. These data suggest that proper developmental regulation and surface localization of HRGPs in ECM were essential for the embryo development and plant regeneration. Future studies should be devoted to the molecular identification and cloning of HRGPs involved in banana somatic embryogenesis.

## Methods

### Plant material

Embryogenic cell suspension (ECS) of 'Yueyoukang 1' (*Musa *spp. AAA) and non-embryogenic cell suspension (NECS) of 'Baxijiao' (*Musa *spp. AAA) were cultured in ZZl medium [47], which is 1/2MS-based [48] and supplemented with 5 μM 2, 4-dichlorophenoxyacetic acid (2, 4-D), 1 μM zeatin and 10 mg/L ascorbic acid. The pH of this medium was adjusted to 6.0 prior to autoclaving. The cultures were incubated at 28 ± 2°C under cool-white light (20 μmol m^-2 ^s^-1^) on a shaker at 90 rpm and sub-cultured at 7 d intervals. The ECs in the ECS were inoculated on RD1 embryo regeneration medium [47] for the development of somatic embryos.

### Monoclonal antibodies and immunofluorescence labelling methods

The monoclonal antibodies JIM11and JIM20, originally described by Smallwood et al. and Knox et al. [20,21], recognize specific arabinosylation epitopes in HRGPs such as extensins and *Solanaceous *lectins. For immunolocalization of HRGPs, ECs and NECs were collected 7 days after the last subculture as well as 5-weeks-old regeneration material on RD1 medium (including somatic embryos at different stages). They were fixed in 3.7% (v/v) formaldehyde in stabilizing buffer MTSB [50 mM piperazine-N, N'-bis(2-ethanesulfonic acid) (PIPES), 5 mM MgSO_4_×7H_2_O, 5 mM ethylene glycol-bis(2-aminoethylether)-*N, N, N', N'*-tetraacetic acid (EGTA), pH 6.9] for 1 h at room temperature, dehydrated in a successive ethanol series (30%, 50%, 70%, 90%, and 100%) and embedded in Steedman's wax [49]. Thin sections (8-10 μm) were placed on microscope slides (Carl Roth GmbH & Co KG). Sections were de-waxed and rehydrated in a successive ethanol series (100%, 90%, 70% and 50%), blocked in phosphate-buffered saline (PBS) supplemented with 50 mM glycine and 2% bovine serum albumin (BSA). To detect the presence and distribution of HRGPs, tissue sections were labelled with primary monoclonal antibodies JIM11 and JIM20 respectively at 4°C overnight (Plant Probes, UK). The primary antibodies were diluted 1:20 in PBS containing 1% BSA. After washing in PBS three times (each for 5 min), the sections were incubated in anti-rat IgG-FITC diluted 1:20 in the same buffer for 1 h at room temperature. After labelling, the slides were washed with PBS (three times, each for 10 min) and stained with 4'-6-diamidino-2-phenylindole dihydrochloride (DAPI). After several rinses with PBS, the sections were stained with 0.01% of toluidine in PBS for 10 min to quench tissue autofluorescence. Finally, the sections were rinsed with PBS (three times, each for 10 min) and mounted in anti-bleach medium before observation (sealed with nail varnish and stored at -20°C). Sections probed only with secondary antibodies were used as controls. There were minimum 5 slides for each antibody. Fluorescence was examined with an Axiovert 35 epifluorescence microscope (ZEISS, Germany). Exposure time was 10000 ms or 2500 ms for lower and higher magnifications, respectively.

### Western blot analysis

ECS and NECS (7 days after the last subculture), somatic embryos at globular stage (cultured on RD1 medium for 3 weeks, incubated at 24°C) and somatic embryos at late stages (cultured on RD1 medium for 5-6 weeks, incubated at 28°C) were collected for the experiments. Cells and tissues (0.3-0.4 g) were ground into fine powder in the presence of liquid nitrogen. Proteins were extracted using 0.7 ml extraction buffer [100 mM Tris, 900 mM sucrose, 10 mM ethylene diamine-tetra-acetic acid (EDTA), 100 mM KCl and 0.4% (v/v) β-mercaptoehtanol, pH 8.8] and 0.7 ml of Tris-saturated phenol (pH 8.8). After centrifugation at 8000 rpm (4°C, 5 min), the supernatant was collected for protein precipitation. The proteins were precipitated by the addition of five volumes of 0.1 M ammonium acetate (in 100% methanol) to the phenol phase, and left at -20°C overnight. Subsequently, the precipitate was centrifuged at 16,000 g at 4°C for 20 min. This precipitate was dissolved in rehydration buffer [8 M urea, 2 M thiourea, 2% CHAPS, 2% Triton X-100, 50 mM 1,4-dithiothreitol (DTT)]. Samples were boiled at 96°C for 5 min and the proteins were separated on 10% sodium dodecyl sulfate-polyacrylamide gel electrophoresis (SDS-PAGE) gels. Proteins were transferred to a polyvinylidene diﬂuoride (PVDF) membrane in a wet tank unit (Bio-Rad) at 60 V by using blot buffer (16 mM Tris-base, 120 mM glycine, 1% SDS, 10% methanol) for 2 hours. PVDF-membrane blots were blocked in TBST buffer (10 mM Tris-base, 150 mM NaCl, 0.1% Tween-20, pH 7.4) containing 4% (w/v) milk powder and 4% BSA for 1 h, followed by labeling with the primary monoclonal antibodies, JIM 11 and JIM20, both diluted 1:200 in TBST buffer containing 1% (w/v) BSA at 4°C overnight. After three rinses with TBST for 10 min, blots were probed with the secondary antibody, a peroxidase-conjugated anti-rat IgGs (Sigma) used at 1:2000 dilution at room temperature for 1.5 h. Protein size markers (Sigma) were 170, 130, 95, 72, 55, 43, 34, 26, 17 and 11 kDa, respectively.

### Plant regeneration via somatic embryogenesis

Plant regeneration through somatic embryogenesis in banana was carried out as described by Xu et al. [50] with slight modification. The ECs of 'Yueyoukang 1' were inoculated on Petri dishes containing RD1 embryo regeneration medium [47] containing full strength MS salts, MS vitamins, 10 mg l^-1 ^ascorbic acid and 100 mg l^-1 ^myo-inositol, for the development of somatic embryos. The cultures were incubated at 28 ± 2°C in the dark. Five weeks later, the regenerated material was weighed and sampled, and transferred to new Petri dishes on top of pre-wetted and pre-weighed Whatman filter papers containing RD2 medium [47] containing full strength MS salts, MS vitamins, 10 mg l^-1 ^ascorbic acid, 100 mg l^-1 ^myo-inositol and 1 μM 6-benzyladenine for further maturation and development of somatic embryos. After 4 weeks of culture on RD2 medium, the weight of the cultures on RD2 medium was evaluated. Then, a representative sample was again weighed and transferred to Petri dishes containing REG medium [47] containing full strength MS salts, MS vitamins, 10 mg l^-1 ^ascorbic acid, 100 mg l^-1 ^myo-inositol, 1 μM indole-3-acetic acid and 1 μM 6-benzyladenine for further development into rooted plants. Finally, weighed samples from the material cultured for four weeks on REG medium were transferred onto rooting and/or shooting medium (MS-based and supplemented with 0.5 μM indole-3- butyric acid, and 1.1 μM 1-naphthylacetic acid). Culture conditions were shifted to 26 ± 2°C and a 16-h photoperiod (50 μmol m^-2 ^s^-1^) after the transfer of embryo masses to the RD2 medium. The number of regenerated plants in every Petri dish was counted.

### Treatment with 3,4-DHP and immunomodulation with JIM11 antibody

The effects of 3,4-DHP and immunomodulation by JIM11 antibody on the embryonic growth as well as regeneration and germination capacities of embryos were examined. Hyp synthesis was inhibited by 200 μM of 3,4-DHP (Sigma), which was added to the somatic embryo regeneration medium RD1. The plant regeneration protocol of 3,4-DHP-treated samples was the same as described above. There were four replicates in each treatment, and about 0.05 g of ECs was inoculated onto RD1 medium in each replicate. Meanwhile, the expression of JIM11 antigen in five weeks old embryos maintained on RD1 medium supplemented with 3,4-DHP was monitored by immunofluorescence microscopy as described above. For immunomodulation, the embryos regenerated on RD1 medium (five weeks old) were treated with JIM11 antibody (diluted 1:20 in the RD1 liquid medium) on a shaker at 120 rpm for two hours. Samples treated only with RD1 liquid medium for 2 h were used as controls. Plant regeneration protocol of treated samples was the same as described above. There were three replicates in each treatment, and about 360 embryos in each replicate. During the whole plant regeneration process, the samples were regularly observed under a Leica binocular microscope and photographed when necessary.

## Abbreviations

3,4-DHP: 3,4-dehydro-L-proline; AGPs: arabinogalactan-proteins; BSA: bovine serum albumin; DAPI: 4'-6-diamidino-2-phenylindole dihydrochloride; DTT: 1,4-dithiothreitol; ECM: extracellular matrix; ECS: Embryogenic cell suspension; ECs: Embryogenic cells; EDTA: ethylene diamine-tetra-acetic acid; EGTA: ethylene glycol-bis(2-aminoethylether)-*N, N, N', N'*-tetraacetic acid; HRGPs: hydroxyproline-rich glycoproteins; Hyp: hydroxyproline; ISG: inversion-specific glycoprotein; NECS: non-embryogenic cell suspension; NECs: non-embryogenic cells; PBS: phosphate-buffered saline; PIPES: piperazine-N, N'-bis(2-ethanesulfonic acid), sodium salt; PRPs: proline-rich proteins; PVDF: polyvinylidene diﬂuoride; SDS-PAGE: sodium dodecyl sulfate-polyacrylamide gel electrophoresis; THRGPs: threonine-hydroxyproline-rich glycoproteins.

## Authors' contributions

JS and CX planned experiments, CX and CB performed experiments, CX, JS, TT and CB analyzed data and prepared data presentation, JS, CX, TT and DM wrote the manuscript. All authors read and approved the final manuscript.
